# Dynamic Behavior of Submerged Cylindrical Shells Under Combined Underwater Explosion, Bubble Pulsation, and Hydrostatic Pressure

**DOI:** 10.3390/ma18040818

**Published:** 2025-02-13

**Authors:** Ruyi Fan, Gaojian Lin, Hang Zhang, Longfei Zhang, Weifu Sun

**Affiliations:** 1 State Key Laboratory of Explosion Science and Safety Protection, School of Mechatronical Engineering, Beijing Institute of Technology, Beijing 100081, China; 2 Designing Institute of Hubei Space Technology Academy, Wuhan 430048, China; 3 School of Civil Engineering, Southeast University, Nanjing 211189, China; 4 Advanced Technology Research Institute, Beijing Institute of Technology, Jinan 250307, China

**Keywords:** underwater explosion, bubble pulsation, hydrostatic pressure, cylindrical shell, numerical simulation

## Abstract

Understanding the dynamic response of cylindrical shells subjected to underwater explosion is crucial for designing safe underwater vehicles, especially in deep-water environments where the shell structures are prestressed by hydrostatic pressure. The complex combination of external loading crossing different temporal scales—from underwater explosive shock waves to bubble pulsation and hydrostatic pressure—results in a synergic damaging effect on the target structures. In this work, the dynamic responses and buckling failure mechanisms of deeply immersed (≥1300 m) cylindrical shells subjected to underwater explosion were investigated through a numerical approach using the finite element method. A convenient and reliable routine for imposing hydrostatic pressure in the Coupled Eulerian–Lagrangian model was developed and validated. Three-dimensional models, composed of spherical charges and shell targets under deep-water conditions, were established to reveal the influences of key factors, including explosion depth and explosion distance, on the failure modes. The results show that the numerical models presented in this work are capable of simulating the complex synergic effect of hydrostatic pressure, the bubble pulsation process, and shock waves on the failure mechanisms of deeply immersed cylindrical shells. This work could provide valuable guidance for the design of safer deep-water marine structures.

## 1. Introduction

High-value marine structures, such as the pressure hull of unmanned underwater vehicles, may encounter threats from deep-water explosion events. It is of great significance to study the dynamic response of cylindrical shells under the condition of great water depths for the effective design of explosion protection for deep-sea vehicles. Due to the different propagation media, the energy release process of trinitrotoluene (TNT) underwater explosions is obviously different from that of air explosions. The phenomena of underwater explosions (UNDEX) include the propagation of explosion shock waves, the generation and pulsation of bubbles, the rupture of bubbles, and the high-speed water jet formed after the rupture of bubbles, resulting in local damage to the ship structure [1,2]. In the process of an underwater explosion, the energy released by the shock wave and bubble pulsation is roughly equal, but bubble pulsation loading is the main factor influencing cylindrical shell deformation under the near field of underwater explosions. Bubble pulsation loading accounts for about 70% and the shock wave loading of underwater explosions accounts for only 30% [3,4,5].

In the current literature, the dynamic responses of shell structures under the action of shallow water explosions and near-surface explosions have been extensively studied [6,7,8,9]. After World War II, Cole et al. [10,11] conducted a large number of underwater explosion experiments and summed up a series of theoretical formulas which provided the basis for subsequent underwater explosion research. However, because experiments equivalent to actual underwater explosions are expensive and difficult to observe, researchers usually use small equivalent explosives to carry out underwater explosion experiments in explosion pools or explosion water tanks, obtaining shock wave and bubble pulsation loading data through pressure sensors, high-speed photography, and other technologies. Brett et al. [12] studied the acceleration, velocity, and deformation of underwater steel cylinders under near-field explosion conditions using a scaled experiment. Zhang et al. [13] studied the dynamic response mechanism of shipboard equipment under underwater explosion conditions by changing the parameters, such as charge position, angle of attack, and equipment installation position, which provided a certain reference for subsequent ship design. The effects of boundary conditions on the pulsation phenomenon and motion process of underwater explosion bubbles were studied by Cui et al. [14] through small-charge underwater explosion experiments. Ren et al. [15] used an airbag composed of oxygen and acetylene instead of TNT to perform an underwater explosion experiment and explored the buckling law of stiffened cylindrical shells under explosive impact loading. Compared with the underwater explosion of explosives, this method significantly increases the action time of the impact load, but it is still very different from the real explosion experiment. Gannon [16] carried out experiments and numerical simulations on the near-field explosion of underwater cylinders. The failure mode obtained through these simulations is in good agreement with the experimental data. Gao et al. [17] studied the deformation of a shell-water-shell structure caused by underwater explosion loading through experiments and numerical simulations. Li et al. [18] introduced a volume acceleration model to determine the initial state of bubble motion in shallow water explosions. They further developed a subroutine within the framework of the MSC.DYTRAN software to describe the initial and boundary conditions of the flow field. Based on the interaction between bubbles and free surfaces, the dynamic behavior of bubbles near free surfaces is simulated and analyzed. Mao et al. [19] studied the dynamic response of pressurized cylindrical shells with different internal pressures and wall thicknesses under the action of a near-field underwater explosion, and corresponding numerical simulations were carried out using LS-DYNA. Li et al. [20] analyzed the damage mechanism of double-layered cylindrical shells. They found that increasing the thickness of the shell and the water interlayer can effectively reduce the impact of the explosion shock wave and the water jet. However, the scale experiments are based on the similarity theory, but the accuracy of the similarity theory is still controversial, especially when it comes to complex structures and complex physical processes, where the accuracy of similar relations may be affected.

Compared to underwater explosions in shallow-water regions, deep-water environments with high hydrostatic pressure have a great influence on the propagation of underwater explosion shock waves, the formation of water jets, and the dynamic response of shell structure targets. The structural response mechanisms of cylindrical shells obtained in shallow water explosions could not be simply extended to deep-water explosions. Gao et al. [21] established a one-dimensional wedge-shaped Euler grid numerical model, using AUTODYN software to simulate underwater explosions at different depths and analyzing the influence of water depth on the peak overpressure and energy of underwater explosion shock waves. Their results show that the peak overpressure and shock wave energy decrease with the increase in water depth, while the percentage of the decrease in peak overpressure with increasing water depth is very small. Xiao et al. [22] introduced an explosion experiment in the deep-sea area of the South China Sea and measured the peak pressure of the shock wave, which was in good agreement with the predicted value from previous studies. However, since the deep-sea full-scale experiment has some shortcomings, such as huge cost, long test duration, and difficulty in observation, it is a feasible method to study deep-water explosions by pressurizing the pressure vessel to obtain hydrostatic pressure equivalent to depths of hundreds of meters. Ma et al. [23] simulated deep-water hydrostatic pressure by increasing the air pressure on the water surface in a pressure vessel. Based on this, the equivalence of the simulation experiment for deep-water explosion with a small equivalent is verified, and the correlation attenuation coefficient between pulsation period and water depth is fitted. Gupta et al. [24] studied the dynamic instability mechanism of metal cylindrical shells caused by underwater explosion loading through experiments. The magnitude of the hydrostatic pressure has a great influence on the dynamic buckling of the structure. When the hydrostatic pressure is small, elastic vibrations can be observed more easily, while larger initial pressure causes the permanent deformation of the shell.

Li et al. [25] used a cylindrical explosive chamber filled with water to carry out an explosion experiment, putting forward an optimal design theory of a water-filled containment vessel. Liang et al. [26] conducted a deep-water explosion experiment in a pressure vessel. Their research results show that the shape of the charge influences the initial shape of the bubble. Bubbles tend to become spherical in shallow waters; however, in deep water (>500 m) bubbles do not become spherical during the first bubble pulsation. Due to the limitations of its materials, the pressure vessel can only carry out underwater explosion experiments at several hundred meters, and the explosive equivalent is limited as well.

Because of the difficulty in conducting full-scale deep-water experiments, and even small-scale experiments using pressure vessels at water depths over 800 m, numerical simulations could be an effective approach to investigate structural responses to deep-water explosions. Currently, simulations of underwater explosions mainly use the structural–acoustic coupling method, which is incapable of capturing the effects of bubble pulsation [27,28,29,30,31]. So far, the explosion dynamic response mechanisms of cylindrical shell structures, under the combined loading of hydrostatic pressure, explosion shock waves, and bubble pulsation pressure, remain largely unexplored.

Although the current research has made remarkable progress in underwater explosions, there is still a relative lack of research on the dynamic response of metal cylindrical shells to explosions under deep-water conditions. Due to the limitations of experimental equipment, the existing research on underwater explosions is mainly concentrated on shallow water (less than 600 m). In this research, underwater explosions in deep-water environments were modelled using the Coupled Eulerian–Lagrangian (CEL) method. The aim of this paper was to reveal the failure mechanisms of metal cylindrical shells under the combined action of hydrostatic pressure, shock wave loading, and bubble pulsation loading, and to explore the influences of different water depths and different explosion distances on the damage efficiency of underwater explosions against cylindrical shells. The findings of this work about the damage mechanisms of thin-walled marine structures under deep-water explosion conditions can guide the improvement of the structural design and impact resistance of marine equipment such as deep-water oil wells, submarine pipelines, and unmanned underwater vehicles.

This article is organized as follows: Section 2 describes the details of the finite element model developed in this work and the manner in which hydrostatic pressure is loaded. In Section 3, the numerical model is verified by comparing the shock wave loading with an empirical formula, the bubble pulsation period with the maximum radius of the bubble, and the experimental results. Additionally, the loading condition of the shell under hydrostatic pressure is verified, ensuring the prestressed state of the shell at great water depths. In Section 4 gives the effects of different explosion parameters—namely, explosion distance and explosion depth—on the dynamic response mechanism of the shell. are given. In Section 5, the main conclusions from this work are given.

## 2. Numerical Simulation Method

### 2.1. Numerical Simulation Models

**Figure 1:**
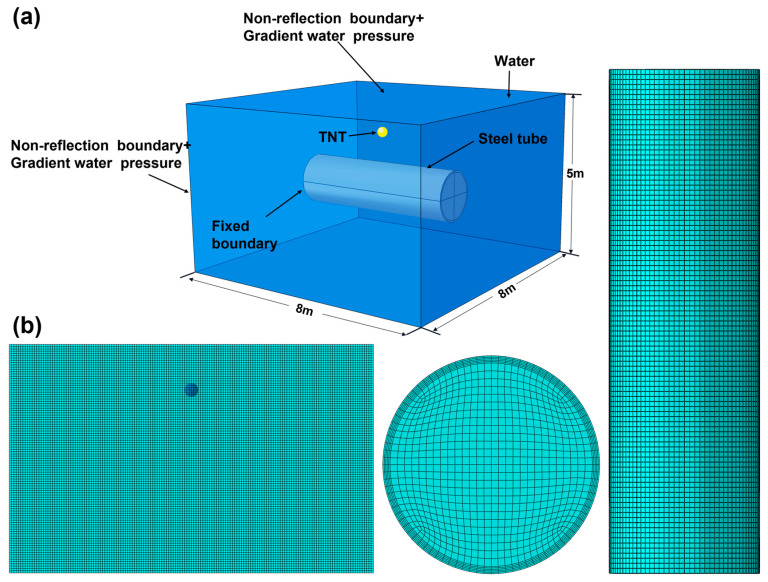
The numerical model used in this work: (**a**) the geometric model and boundary conditions; (**b**) the mesh of the water domain and the cylindrical shell.

As shown in Figure 1a, in this study, a three-dimensional model was established to simulate an underwater explosion in a deep-water environment using ABAQUS software (6.14). Water and TNT were modeled using Eulerian elements, and their material information were discretized into the Eulerian domain using the volume fraction method. The cylindrical shell adopts Lagrangian elements. The meshing of the model is shown in Figure 1b. The water domain is a cuboid of 8 m × 8 m × 5 m, and the Z direction is vertical. The fluid domain is described by multi-material Euler elements. The radius, thickness, and length of the cylindrical shell are 0.75 m, 0.05 m, and 5 m, respectively. Both ends of the shell are constrained by displacement, except for axial displacement. Considering the efficiency and accuracy of the simulations, the mesh of the shell is refined along the thickness of the shell wall, with a size of 0.01 m. The uniform mesh is used in the water domain, and the mesh size is 0.05 m.

However, due to the limitations of the performance of computing equipment, it is impossible to further refine the grid of the water domain. The volume fraction method performs a Boolean operation on the Euler body and its intersecting reference volume and then generates a discrete scalar field corresponding to the unit-scale information. Therefore, if TNT with a smaller weight is used, the Boolean operation causes the TNT to become a polyhedron rather than a sphere, which will cause a large weight error of explosives, affect the explosion parameters in subsequent research, and increase the deviation from experimental results. Therefore, the weight of TNT is determined by its volume, which is 23.891 kg for a radius of 0.15 m in the simulations. The TNT charge is positioned 4 m away from the side of water domain, 1 m away from the top, and 4 m away from the bottom.

### 2.2. Material Definition

The water body adopts the US-UP equation of state model, which can simulate incompressible viscous and inviscid laminar flows governed by the Navier–Stokes equation of motion. The equation of state assumes that pressure is a function of the density and internal energy of the unit mass [32]. It is written as follows:

(1) $$p-p_{H}=\Gamma \rho \left(E_{m}-E_{H}\right)$$

where p_H_ is the Hugoniot pressure, Γ = Γ_0_ρ_0_/ρ is the Mie–Grüneisen coefficient, ρ_0_ is the reference density, E_H_ is the Hugoniot energy, and E_m_ is the specific internal energy. The relation between E_H_ and p_H_ is expressed as follows:

(2) $$E_{H}=\frac{p_{H}\eta }{2\rho_{0}}$$

where the nominal volumetric compressive strain is defined as η = 1 − ρ_0_/ρ. The Hugoniot pressure p_H_ is generally expressed as follows:

(3) $$p_{H}=\frac{\rho_{0}{c_{0}}^{2}\eta }{{\left(1-s\eta \right)}^{2}},$$

It is assumed that the shock velocity U_s_ and the particle velocity U_p_ are linearly related, and U_s_ and U_p_ meet the equation U_s_ = c_0_ + sU_p_. The Mie–Grüneisen equation can be written as follows:

(4) $$p=\Gamma_{0}\rho_{0}E_{m}+\frac{\rho_{0}{c_{0}}^{2}\eta }{{\left(1-s\eta \right)}^{2}}\left(1-\frac{\Gamma_{0}\eta }{2}\right)$$

where c_0_ is the speed of sound propagation in the medium. The parameter settings are shown in Table 1.

The equation of state for TNT explosives can be described by the standard Jones–Wilkins–Lee (JWL) equation of state:

(5) $$p=A\left(1-\frac{\omega }{R_{1}V}\right)e^{-R_{1}V}+B\left(1-\frac{\omega }{R_{2}V}\right)e^{-R_{2}V}+\frac{\omega E_{0}}{V}$$

where p is the pressure of detonation products, E_0_ is the internal energy per unit volume, A, B, R_1_, R_2_, and ω are constants, which are obtained from the explosion experiment, and V is the relative volume. The TNT parameters are shown in Table 2. For the shell, 45# steel is used, which can be described by the Johnson–Cook constitutive model. It is a high-quality carbon structural steel with a carbon content of 0.45%, so it is named 45# steel. This kind of steel is widely used in the manufacture of mechanical parts because of its good comprehensive mechanical properties. The parameters are shown in Table 3 [33].

### 2.3. Numerical Simulation Method

Before the underwater explosion loading acts on the shell, the shell is in a prestressed state due to the existence of hydrostatic pressure. Therefore, when studying deep-water explosions, it is necessary to consider the response of the shell to hydrostatic pressure. To realize the prestressed state of the cylindrical shell under a predetermined water depth and stable loading of hydrostatic pressure, a three-step simulation scheme is developed in this study. In the first step of the analysis, uniform pressure is applied to the cylindrical shell to realize a prestressed state at the predetermined water depth. The second step is to carry out the stable loading of the hydrostatic pressure in the water domain, during which the spherical charge and the cylindrical shell are completely fixed as rigid bodies by displacement constraints. After this step, the hydrostatic pressure of the water domain satisfies the pressure condition in the predetermined water depth environment, and the shell is not affected by the impact of the fast-rising hydrostatic pressure. In the third step of the analysis, the displacement constraints of the explosive and the cylindrical shell are removed, and the explosive is detonated by setting the delayed initiation time.

Boundary conditions have a great influence on the simulation results. The bubble pulsation characteristics of the underwater explosion in free field are in good agreement with Cole’s classical theory. The fixed boundary conditions cause the explosion shock wave to be reflected when it reaches the wall. The reflected shock wave acting on the bubble affects the bubble pulsation process [14,34]. Due to the exponential attenuation of the underwater explosion shock wave in the water, 48% of the energy will be dissipated in a medium with a radius of 25 times the explosive radius. Therefore, the influence of the boundary can be reduced by increasing the ratio of the water domain size to the maximum bubble size. In the subsequent simulations in this paper, the water boundary condition is set to a normal velocity constraint in the shell pressurization stage. In the stage of water pressurization and underwater explosion, the non-reflective outflow condition can be used for the water domain. The non-reflective boundary condition is selected to allow the material to flow out of the Euler region, and additional normal and tangential pulling forces are introduced on the regional boundary which are proportional to the normal and tangential components of the boundary velocity. These boundary damping constants are selected to minimize the reflection of expansion wave and shear wave energy back to the finite element mesh. This condition cannot provide perfect energy transmission except in the case of plane body wave orthogonal impact on the boundary of an isotropic medium. However, for most real-world cases, it usually provides acceptable modeling.

After the explosive detonation, the explosion shock wave propagates outward. Part of the shock wave will be reflected into the flow field when it reaches the shell wall. The superposition of reflected and incident waves can form a low-pressure area. When the pressure in the area is lower than the vapor pressure of water, the water will turn into the gas phase due to cavitation, and a cavitation zone will be formed above the shell. To simulate the cavitation effect, it is necessary to embed the cavitation model in the CEL method. In the truncation model, when the pressure in the water area is below a certain critical value (usually the saturated vapor pressure), the pressure in the area is set to take the critical value. Therefore, the truncation water pressure is set to zero in the simulation process.

## 3. Validation of the Numerical Model

### 3.1. Validation of Shock Wave Loading

Researchers have conducted a lot of studies on pressure changes in flow fields caused by underwater explosions. Zamyshlyaev et al. [35] studied underwater explosion loading through a large number of experiments, and divided underwater explosion loading into five stages: exponential attenuation, reciprocal attenuation, bubble expansion, shrinkage, and pulsating pressure. Li et al. [36] simplified the pressure change process of underwater explosions and gave the simplified formula for pressure change in different stages. Due to the use of the cavitation truncation model, this work only verifies the attenuation stage of the explosion shock wave and the pulsating pressure stage. Zamyshlyaev’s formulas are as follows:

(6) $$P(t)=P_{m}\cdot e^{-t/\theta },\;t<\theta$$

(7) $$P(t)=P_{m}\cdot 0.368\cdot \frac{\theta }{t}\cdot \left[1-{\left(\frac{t}{t_{p}}\right)}^{1.5}\right],\;\theta \leq t<t_{1}$$

(8) $$P(t)=P*\cdot \left[1-{\left(\frac{t}{t_{p}}\right)}^{1.5}\right]-\Delta P,\;t_{1}\leq t\leq t_{p}$$

(9) $$P(t)=P_{m1}\cdot e^{-(t-T{)}^{2}/{\theta_{1}}^{2}},\;T-t_{2}\leq t\leq T+t_{2}$$

in which

$$P_{m}=\left\{\begin{array}{c} 4.41\times 10^{7}{\left(\frac{\omega^{1/3}}{R}\right)}^{1.5},\;6\leq \frac{R}{R_{0}}<12\\ 5.24\times 10^{7}{\left(\frac{\omega^{1/3}}{R}\right)}^{1.13},\;12\leq \frac{R}{R_{0}}<240 \end{array}\right.,$$

$$\theta =\left\{\begin{array}{c} 0.45R_{0}\cdot \bar{r}\cdot 10^{-3},\;\bar{r}\leq 30\\ 3.5\frac{R_{0}}{c}\cdot \sqrt{\mathrm{lg}\bar{r}-0.9},\;\bar{r}>30 \end{array}\right.,$$

$$t_{1}=\frac{R_{0}}{c}\left(\bar{r}-m\right),\;\bar{t}=\frac{c}{R_{0}}t,$$

$$P*=7.173\times 10^{8}/\bar{r}\times {\left(\bar{t}+5.2-m\right)}^{-0.87}$$

$$m=11.4-10.6/{\bar{r}}^{0.13}+1.51/{\bar{r}}^{1.26}$$

$$t_{p}=\left(850/\bar{P_{0}}-20/{\bar{P_{0}}}^{1/3}+m\right)\frac{R_{0}}{c}$$

$$\Delta P=10^{5}/{\bar{r}}^{4}\left(5635{\bar{t}}^{0.54}-0.113{\bar{P_{0}}}^{0.15}\cdot {\bar{t}}^{2}\right)$$

$$P_{0}=P_{\mathrm{atm}}+\rho \cdot g{\cdot H}_{0},\;\bar{P_{0}}=P_{0}/P_{\mathrm{atm}},$$

$$t_{2}=3290\cdot R_{0}/{P_{0}}^{0.71},\;\bar{r}=R/R_{0},$$

$$P_{m1}=7.1\times 10^{6}\cdot \frac{\omega^{1/3}}{R_{1}},\;\theta_{1}=20.7\frac{R_{0}}{{P_{0}}^{0.41}},$$

where P_m_ is the peak pressure of the explosion shock wave in Pa; θ is the time decay constant of the shock wave in s; ω is the weight of the spherical charge in kg; R is the distance between the explosion center and the observation point in m; R_0_ is the radius of the spherical charge in m; t_1_ is the time when the shock wave propagates to the observation point in s; t_p_ is the positive pressure time of shock wave in s; P_0_ is the hydrostatic pressure at the position of the charge in Pa; P_atm_ is atmospheric pressure in Pa; c is the speed of sound propagation in water in m/s; H_0_ is the depth of the explosion center in m; and P_m1_ is the peak value of the secondary pulsation pressure in Pa. The empirical formula for the peak pressure of TNT explosives estimated by Cole without considering the upward movement of the bubble is used. The center point of the second bubble pulsation is the reference point, and R_1_ is the distance from the reference point to the measuring point; $\theta_{1}$ is the time decay constant of the secondary pulsating pressure, and the unit is s.

**Figure 2:**
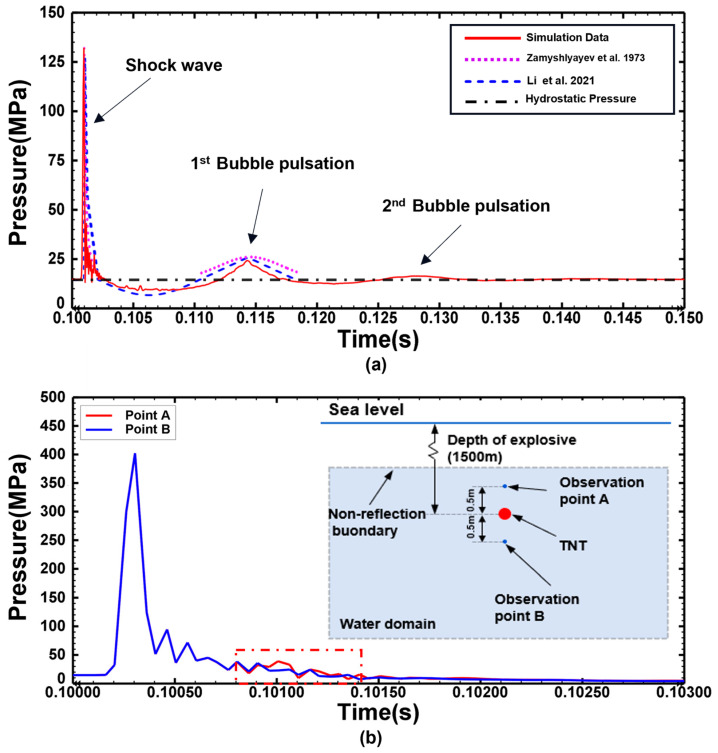
(**a**) Pressure time-history curves at the horizontal distance of 150 cm from the detonation center under the condition of water depth H = 1500 m [35,36]. (**b**) Pressure time–history curve of the flow field when the reflected shock wave acts on the surface of the bubble and at the same distance below the bubble.

To verify the effectiveness of the simulation method, an explosion at a water depth of 1500 m without a shell model is simulated, and the explosion shock wave and hydrostatic pressure are first verified. The pressure time–history curve, extracted at a horizontal distance of 150 cm from the explosion center, is shown in Figure 2a. The hydrostatic pressure in the water domain is fairly stable before the explosive detonation and reaches the hydrostatic pressure of the determined water depth as expected. After the explosive is detonated, the peak pressure of the explosion shock wave and the peak pressure of secondary loading appear successively at the observation point. The simulated peak pressure of the explosion shock wave and bubble pulsation are in good agreement with the theoretical values. Eventually, the pressure at the observation point stabilizes at hydrostatic pressure in the later stages of the simulation.

The non-reflective outflow condition introduces additional normal and shear tractions on the domain boundary that are proportional to the normal and shear components of the velocity of the boundary. These boundary damping constants are chosen to minimize the reflection of dilatational and shear wave energy back into the finite element mesh. Figure 2b shows the pressure change in the flow field when the reflected shock wave acts on the bubble surface and the pressure change at the same distance below the bubble. The pressure change at point B is less affected by the reflected shock wave, and the pressure change at point A fluctuates but roughly coincides with that at point B, indicating that the non-reflective boundary condition greatly reduces the reflection phenomenon of the boundary and meets the needs of subsequent research.

The empirical formulas for the variation in the bubble pulsation period and the maximum bubble radius with water depth in deep water explosions are as follows [23]:

(10) $$R_{m}=3.697\times \frac{\omega^{1/3}}{(H+10{)}^{0.364}}$$

(11) $$T=2.046\times \frac{\omega^{1/3}}{(H+10{)}^{0.83}}$$

where R_m_ is the maximum bubble radius in m; T is the bubble pulsation period in s; ω is the weight of the explosive charge in kilograms; and H is the water depth of the location of the explosion in m.

The simulated value of the bubble pulsation period is 0.0130 s, the theoretical value is 0.0135 s, and the deviation is 3.7%. The simulated value of the maximum radius of the bubble is 0.750 m, the theoretical value is 0.745 m, and the deviation is 0.67%. It can be seen that the bubble evolution process, generated by the underwater explosion simulation, is in good agreement with the bubble pulsation process in the experiment.

**Figure 3:**
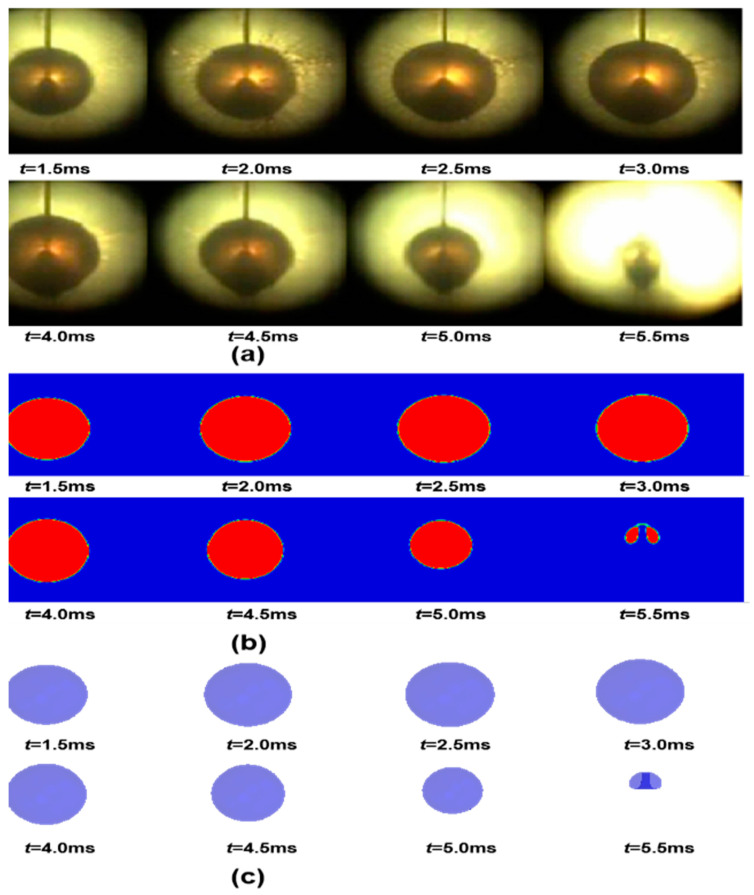
Comparison of bubble calculation results to experimental results [23]. (**a**) Experimental observation diagram of bubble pulsation process; (**b**) Profile view of simulation result; (**c**) Schematic diagram of bubble pulsation process.

The bubble pulsation process and energy release process of an underwater explosion of 1 g TNT at 0.6 MPa pressure are simulated and compared to the experimental values in [23], as shown in Figure 3. Figure 3a shows the bubble motion recorded by high-speed photography during the experiment, while Figure 3b,c show the bubble profile and bubble pulsation process using the same simulation method in this work under the same hydrostatic pressure as the experiment. It can be seen from Figure 3 that the simulation results are in good agreement with the experiments at different stages of bubble movement. In Table 4, the simulated values are compared to the experimental values, in which the deviation for the maximum bubble radius is 2.65% and the deviation for the bubble pulsation period is 6.67%.

Through the comparison of underwater explosion experiments under hydrostatic pressure and the numerical simulation of underwater explosions under great water depths, it can be known that the numerical simulation method has a high simulation accuracy and can meet the needs of subsequent research.

### 3.2. Response of the Shell to Hydrostatic Pressure

The deep-water environment is usually accompanied by huge hydrostatic pressure. For cylindrical shells in the deep sea, when the external pressure increases and reaches its critical value, the shell may change from the static equilibrium state to the critical instability state. For deep-sea facilities such as deep-sea detectors and submersibles, materials need to be able to withstand huge water pressure without deformation or damage to ensure the safety and stability of the equipment. They must also be able to resist the impact of water and the corrosion of sea water, which requires the materials to have excellent impact resistance and corrosion resistance at great water depths. These properties ensure the long-term stability and reliability of materials in deep-water environments to meet a wide range of deep-water operational requirements.

**Figure 4:**
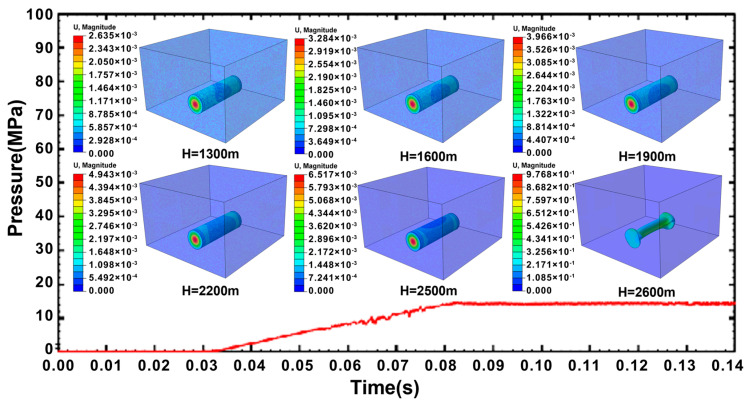
Time-history curve of pressure loading in water without shell structure, and the displacement response of the cylindrical shell to hydrostatic pressure corresponding to different water depths.

By applying uniform pressure, the ultimate load-bearing capacity of the shell can be found, and the following simulated water depth interval is determined. Before the explosive is detonated, the surface wall and both ends of the shell are subjected to the hydrostatic pressure of the predetermined water depth. When the water depth is less than 2500 m, there is no obvious deformation on the surface of the cylindrical shell. The hydrostatic pressure on the surface of the cylindrical shell progressively rises as the depth of the water increases. The shell structure can no longer withstand the pressure when the water depth exceeds 2600 m, and the hydrostatic pressure induces the buckling collapse of the cylindrical shell. An identical cylindrical shell is also used for linear buckling load prediction using the keyword *Buckle. The obtained critical pressure matches the 2600 m water depth. Figure 4 shows the displacement response of the cylindrical shell to hydrostatic pressure when the explosion distance is 225 cm. The result demonstrates that stable loading of hydrostatic pressure could be achieved, and the cylindrical shell does not exhibit any unexpected motion, satisfying the requirement of the subsequent simulations.

### 3.3. Effect of Hydrostatic Pressure on the UNDEX Loading

**Figure 5:**
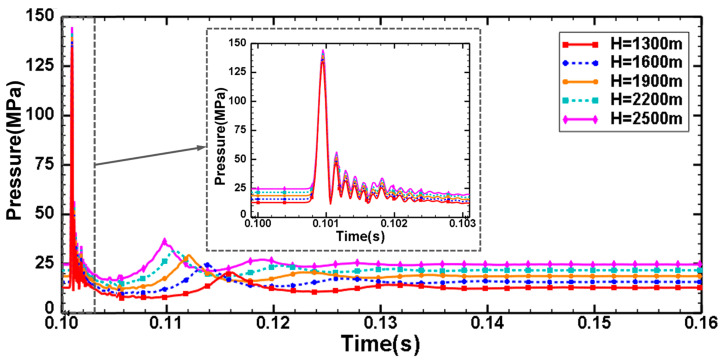
Pressure time–history curves of the points at the horizontal distance 150 cm from the detonation center under the condition of water depth H = 1500 m.

**Figure 6:**
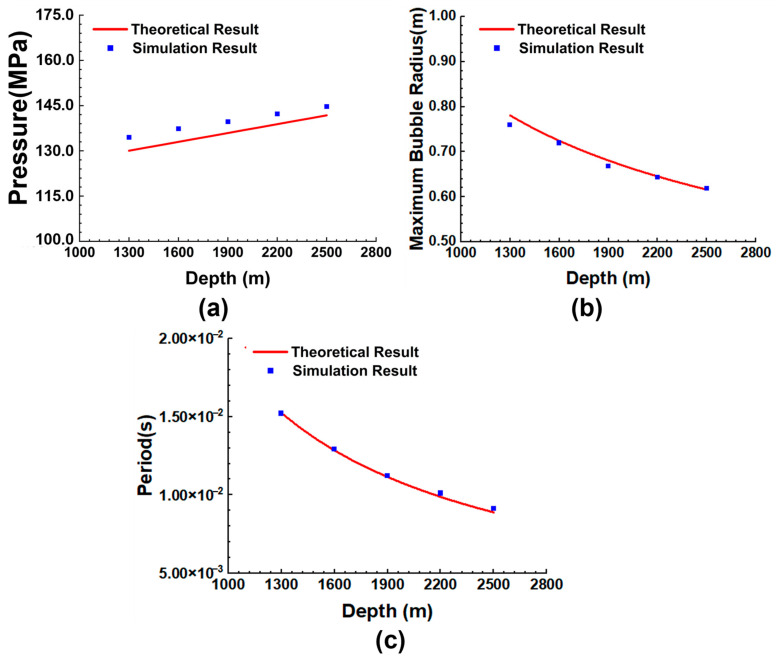
Comparison between theoretical and simulated values of explosion parameters under different explosion depths: (**a**) peak pressure of the shock wave; (**b**) maximum bubble radius; (**c**) bubble pulsation period.

The existence of hydrostatic pressure makes the damage process of underwater explosions very different from that of explosions in air. The results of Gao et al. [21] show that the peak overpressure of shock waves decreases by about 0.5% per kilometer with increasing explosion depth. Figure 5 shows the pressure time–history curves of the points at a horizontal distance of 150 cm from the explosion center, and the hydrostatic pressure before detonation is consistent with the preset water depth. After detonation of the spherical charge, the pressure at the observation points increases rapidly. Due to the small range of water depths studied, there is no significant difference in shock wave peak pressures at different depths, which approximately satisfies the initial hydrostatic pressure difference. The peak value of the shock wave in Equation (6) is only related to the weight of the explosive charge and the explosion distance. It is further explained that the explosion depth has no obvious effect on the shock wave, and that the explosion distance is the main factor influencing shock wave attenuation. Compared to shock wave loading, the effect of water depth on bubble pulsation loading is more significant. With increasing water depth, the bubble pulsation period is further shortened; at the same time, the coupling of bubble pulsation pressure with the shock wave produces a more ideal damage efficiency, and the continuous damage further destroys the structural stability of the shell. As shown in Figure 6a, the peak pressure of the explosion shock wave increases with increasing water depth, and the simulated values are in good agreement with the theoretical values. Figure 6b,c are used to verify the bubble pulsation period and maximum bubble radius. The simulation results show that the increase in the explosion depth accelerates the speed of bubble pulsation, and the bubble radius decreases due to the high hydrostatic pressure. The simulation results are in good agreement with the theoretical values.

## 4. Results and Discussion

### 4.1. Effect of Water Depth

**Figure 7:**
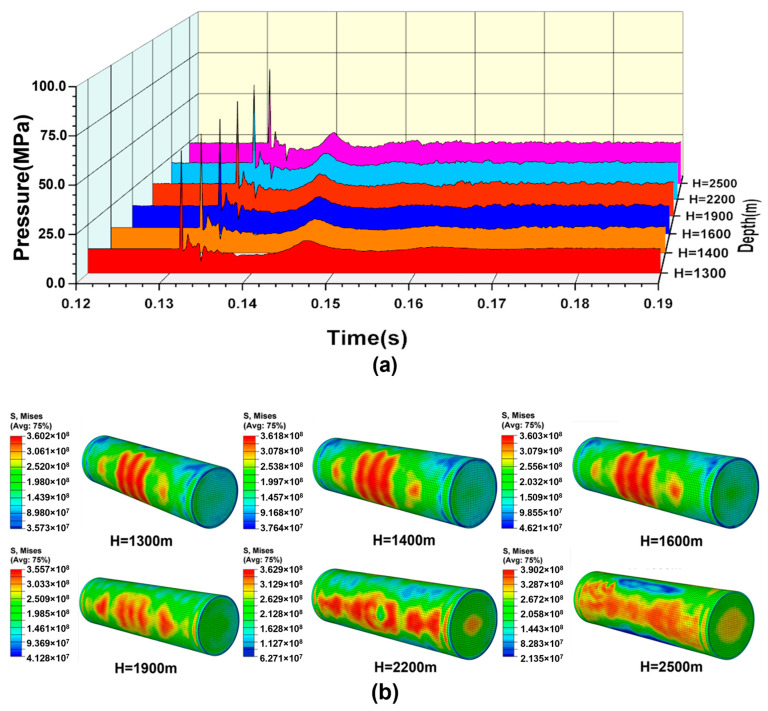

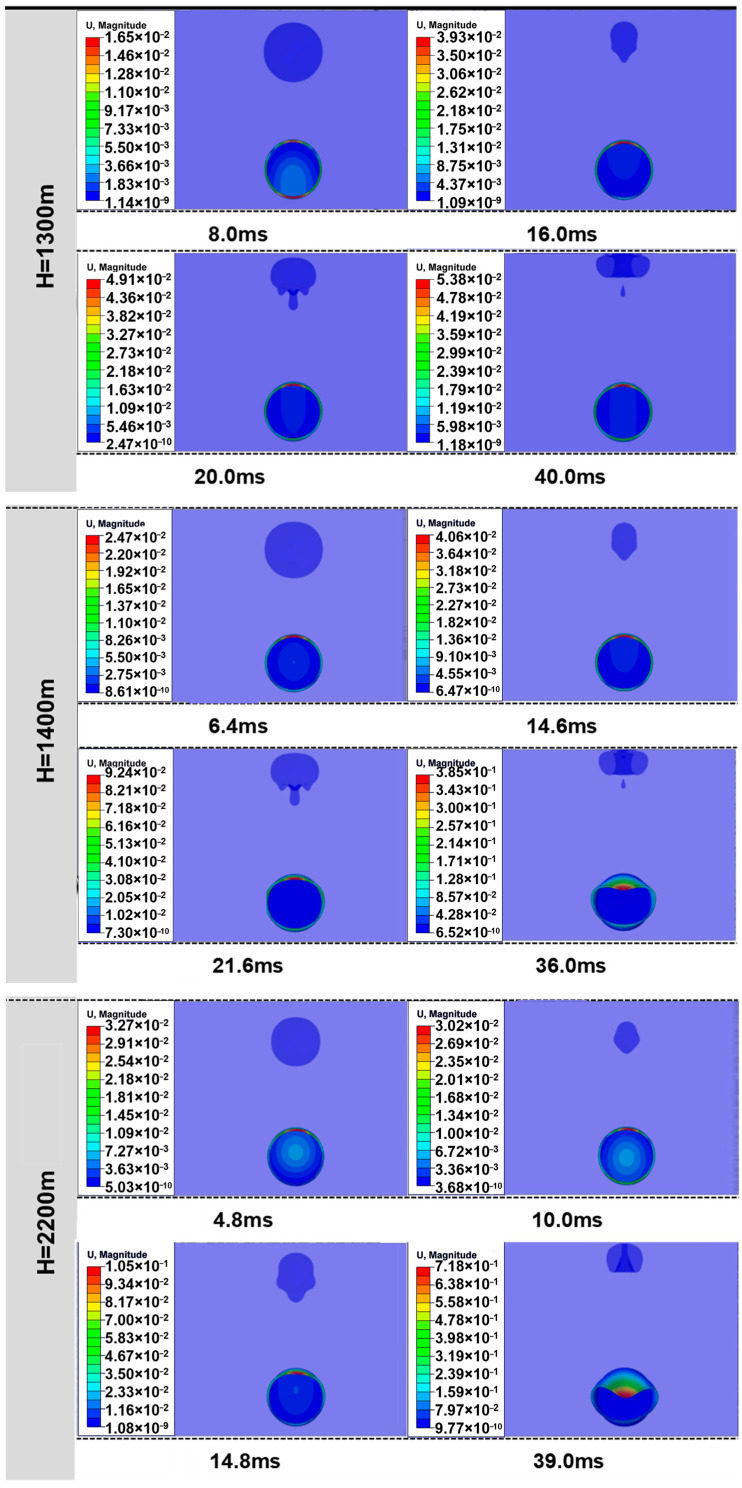

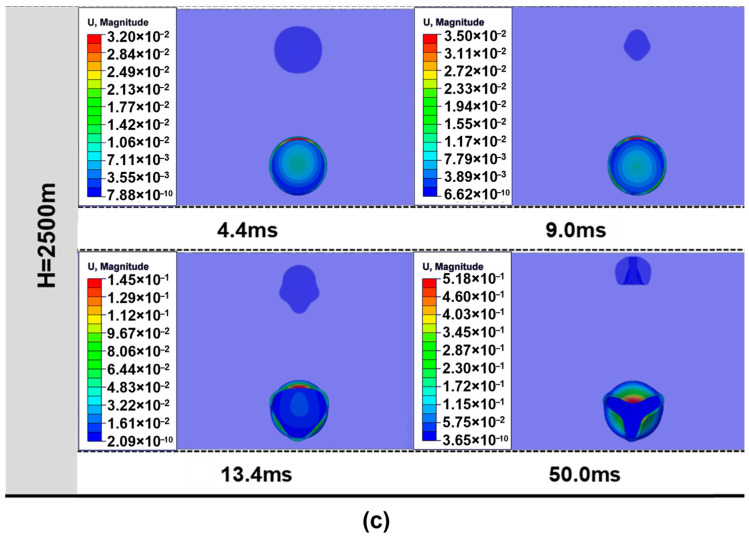
(**a**) Pressure time-history curves of the points at the horizontal distance of 150 cm from the explosion center under the condition of different water depths. (**b**) Dynamic response of the shell with variable water depths after the action of the shock wave. (**c**) Dynamic displacement response of the cylindrical shell obtained from the CEL simulation for the case with different water depths and explosion distance a = 225 cm.

When the explosion distance is 225 cm, the explosion loading response of the cylindrical shell can be studied by changing the water depth. Figure 7a shows the pressure time–history curves of the points at a horizontal distance of 150 cm from the explosion center at different water depths. After the explosion of TNT, the shock wave propagates rapidly. The pressure of the explosion shock wave reaches its peak in a very short time and fluctuates greatly in the attenuation stage. With the increase in water depth, the attenuation effect on the shock wave leads to a slight decrease in the shock wave overpressure. However, under the coupling action of the hydrostatic pressure, the total pressure remains relatively stable. The increase in the explosion depth influences the bubble pulsation period, and the time interval of the secondary loading decreases gradually. At the same time, the increase in the explosion depth leads to an increase in the peak value of the secondary loading pressure, which greatly improves the damage efficiency of underwater explosions. Figure 7b shows the stress-strain state of the shell after explosion shock wave loading at different water depths, and it can be seen that the shell does not undergo large deformation. At this moment, the bubble is still in the stage of expansion.

The dynamic response process of a cylindrical shell under bubble pulsation loading at different water depths is shown in Figure 7c. At a depth of 1300 m, the shell has no buckling deformation under the combined action of shock wave loading and bubble pulsation loading. The bubble undergoes the pulsating process of expansion, shrinkage, and re-expansion, and finally forms an annular bubble. Due to the influence of Bjerknes forces, the contraction process of the lower part of the bubble is affected, resulting in the whole bubble forming a spindle shape. With increasing water depth, the deformation of the shell increases gradually. When the water depth reaches 1400 m, buckling deformation occurs under the secondary loading, and a large deformation occurs at the top of the shell. After the detonation of the TNT, the explosion shock wave propagates outwards rapidly, and a part of the shock wave is diffracted to the back of the cylindrical shell. The action time of the shock wave on the shell surface is quite short, so it quickly passes through, despite the high peak value. As a result, the center of the blast face of the shell does not appear to be largely deformed. Secondary damage of the cylindrical shell occurs under the combined action of hydrostatic pressure and bubble pulsation loading. The action time is much longer compared to the shock wave, which results in the large deformation of the top and middle portions of the cylindrical shell.

With the increase in the explosion depth, the bubble formed by explosion becomes smaller and smaller, and the influence of wall effects on the bubbles decreases gradually. As the water depth reaches 2500 m, the hydrostatic pressure approaches the static load-bearing limit of the shell, and the combined action of the shock wave loading and hydrostatic pressure causes the shell to collapse before the action of the bubble pulsation loading. Additionally, the motion speed of the shell deformation is accelerated by the bubble pulsation loading. The instability mode of the shell is the third-order mode, which is in sharp contrast to the second-order buckling mode under the condition of smaller water depths. This is because the buckling of the shell is triggered by the dynamic loading of the shock wave, with a high loading rate when the water depth is 2500 m. The strong dynamic effect in dynamic buckling will induce a higher buckling mode compared to the quasi-static buckling caused by the bubble pulsation pressure [37,38].

**Figure 8:**
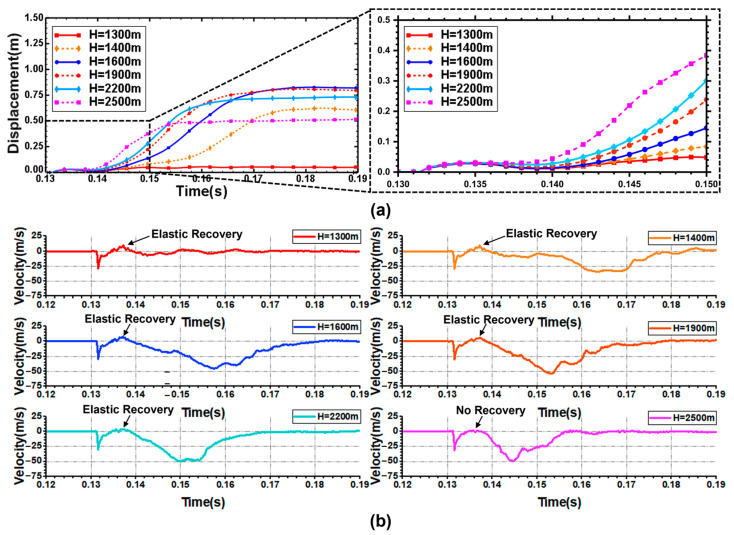
(**a**) Displacement time-history curves at the center points of the blast face and back face of the shell; (**b**) Velocity time-history curves of the center points of the blast face of the shell.

In the bubble shrinkage stage, the bubble is strongly attracted by the structural plane, so the shell will restore a certain elastic deformation, as shown in Figure 8a. This figure shows the displacement curves of the center points of the blast face of the shell when at an explosion distance of 225 cm, under variable water depth conditions. The center point on the blast face of the shell can be accelerated to tens of meters per second after the action of the shock wave when the water depth is 1300 m. However, there is no large deformation because of the short action time. In the process of bubble shrinkage, the elastic deformation of the center point of the blast face of the shell is restored. The water jet formed by bubble expansion hinders further recovery of elastic strain, and then the shell is affected by bubble pulsation loading. In the process of the second bubble pulsation, the deformation of the shell fluctuates continuously and tends to be stable under bubble pulsation loading. Finally, there is no collapse of the shell, and the deformation velocity of the center point of the blast face of the shell approaches 0 m/s.

With the increase in water depth, the recovery of the shell becomes smaller and smaller during the bubble shrinkage stage. This is because the shell is continuously impacted by the combined action of high hydrostatic pressure and the water jet. When the depth reaches 2500 m, the center point of the blast face of the shell produces a relatively static moment in the bubble shrinkage stage, and the deformation neither expands nor recovers. At this time, the hydrostatic pressure can make the shell continue to deform and eventually collapse under the combined action of bubble pulsation.

### 4.2. Effect of Explosion Distance

The explosion distance has a direct effect on both shock wave loading and bubble pulsation loading. By varying the explosion distance between the target shell and the detonation center when the water depth is fixed at 1300 m, the influences of explosion distance on the bubble pulsation process and the dynamic response of the cylindrical shell were studied. Under the condition of a water depth of 1300 m, the theoretical maximum bubble radius is 0.781 m and the theoretical bubble pulsation period is 0.0152 s.

**Figure 9:**
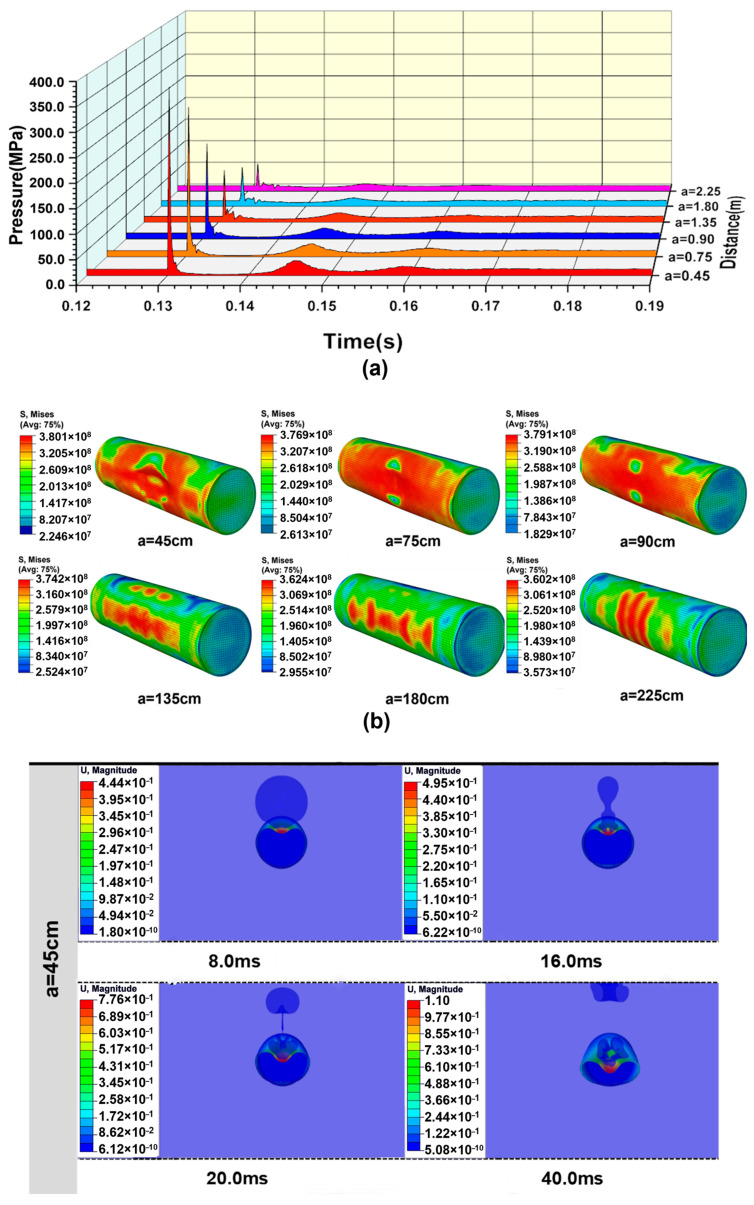

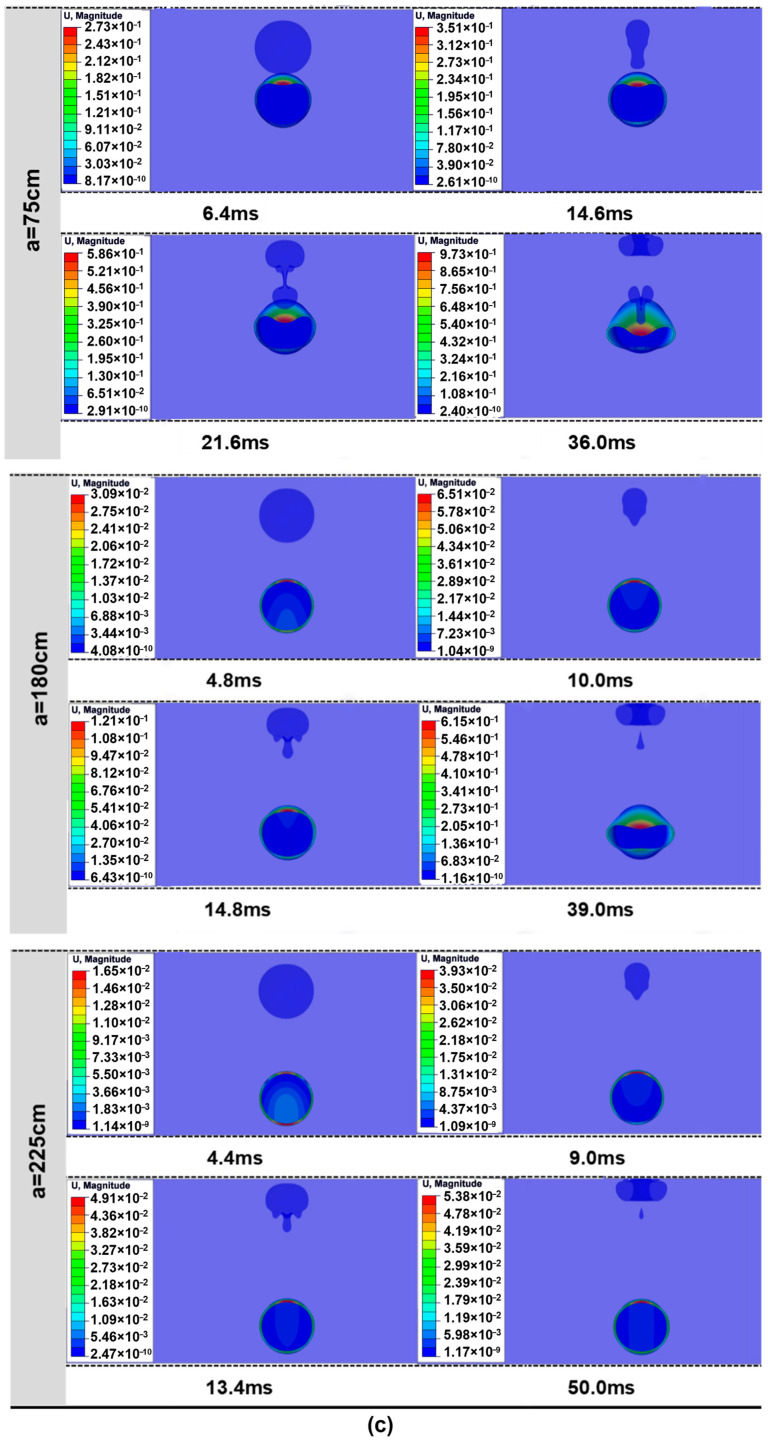
(**a**) Pressure time-history curves of the points on the horizontal plane of the explosion center with the same distance from the explosion center to the shell under the condition of water depth H = 1300 m. (**b**) Dynamic response of the shell with variable explosion distances after the action of the shock wave. (**c**) Dynamic displacement response of the cylindrical shell obtained from the CEL simulation for the case with different explosion distances and explosion depth H = 1300 m.

Figure 9a shows the pressure time-history curves of the points on the horizontal plane of the explosion center with the same distance from the explosion center to the shell under the condition of water depth H = 1300 m. Before the arrival of the shock wave from the explosion, the hydrostatic pressure is relatively stable, and the peak pressure of the shock wave decreases exponentially with the increase in explosion distance. Since the explosion depth is constant, and the bubble pulsation period is consistent, the time interval for the secondary loading is also the same. The increase in explosion distance will also affect the peak overpressure of the secondary loading. When the explosion distance is 45 cm, the shock wave peak value is 376.0 MPa, but when the explosion distance reaches 225 cm, the peak value of the shock wave is only 63.4 MPa.

Generally, if the explosion distance is relatively small, the shell is quickly affected by the shock waveand impacted by the water jet and the direct action of the bubble within a short time. If the explosion distance is relatively large, the shell is first impacted by the rapid explosion shock wave and then impacted by the water jet after a certain period. Since explosion distance has a great influence on secondary loading, when the explosion distance reaches a certain value, the deformation caused by secondary loading prevents the shell from collapsing under hydrostatic pressure. Figure 9b shows the deformation diagram of the target shell after the action of the explosion shock wave and the water jet driven by bubble expansion. It can be seen that when the explosion distance is 45 cm, the bubble loading directly acts on the shell, leading to a large deformation at the top of the shell. The deformation of the shell decreases with increasing explosion distance. When the explosion distance is 225 cm, the target shell has no obvious deformation.

As shown in Figure 9c, when the explosion distance is less than the maximum radius of the bubble, the bubble acts directly on the surface of the shell in the expansion stage, and the top of the shell is deformed greatly. In the bubble shrinkage stage, the bubble is attracted by the shell surface. Finally, a water jet is formed that acts on the shell surface, which makes the shell collapse rapidly under the combined action of hydrostatic pressure. As the explosion distance increases, the influence of the cylindrical shell surface on the bubble pulsation process gradually decreases. When the explosion distance is greater than the maximum bubble radius, the target shell collapses due to the combined effect of hydrostatic pressure and bubble pulsation loading. When the explosion distance reaches 225 cm, it can be considered that the cylindrical shell is in the far-field explosion range, where the combined action of the shock wave and bubble pulsation loading do not trigger structural instability.

**Figure 10:**
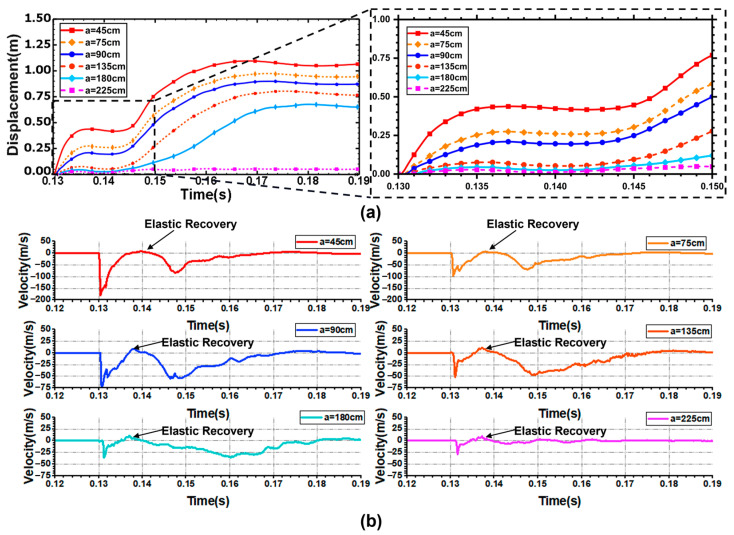
(**a**) Displacement time-history curves at the center points of blast face and back face of the shell; (**b**) Time-history curves of velocity change at the center point of the blast face of the shell.

Figure 10a shows the displacement time–history curve of the center points of the blast face and back face of the shell. During the first bubble pulsation period, the displacement of the center point of the blast face of the shell decreases with the increase in the explosion distance. With the increase in the explosion distance, the attenuation velocity of the explosion shock wave decreases gradually. When the explosion distance is more than 135 cm, the increase in the explosion distance has little effect on the displacement of the shell under the action of the explosion shock wave. Therefore, in the initial stage of the explosion, the displacement curve of the center point on the blast face of the shell does not show much difference. In the process of secondary bubble pulsation, the shell is subjected to the continuous action of bubble pulsation loading and hydrostatic pressure. When the explosion distance reaches 225 cm, the shell does not collapse. Figure 10b shows the change of velocity at the center points of the blast face of the shell. When the explosion distance is 45 cm, the velocity of the center point on the blast face of the shell can reach 192 m/s. When the explosion distance is 225 cm, the shell wall can only reach a speed of tens of meters per second.

### 4.3. Summary of Simulation Results

**Figure 11:**
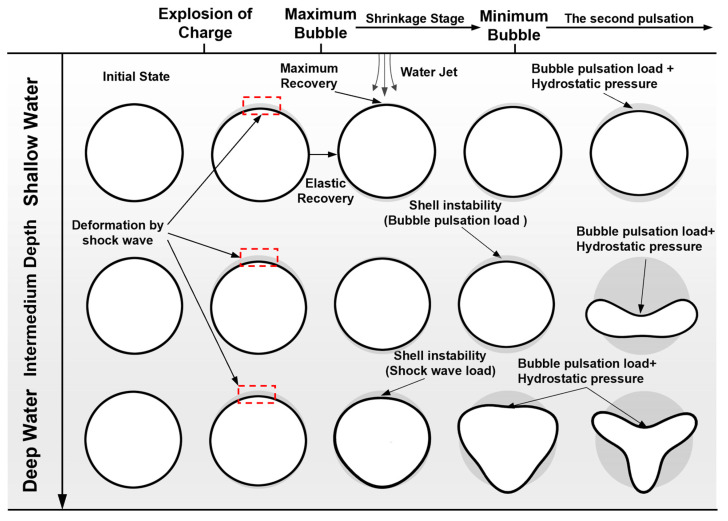
Comparison of the failure mechanisms of the cylindrical shell subjected to underwater explosion loading: under relatively deep-water conditions, where the hydrostatic pressure is not close to the critical buckling pressure of the shell (top); and under extremely deep-water conditions, where the hydrostatic pressure is close to the critical buckling pressure of the shell (bottom).

The failure mechanism of the cylindrical shells subjected to underwater explosions at different explosion depths can be summarized as illustrated in Figure 11. In shallow water, the shell is successively subjected to an explosion shock wave load and a bubble pulsation load. The shell is deformed after the shock wave, and then part of the elastic strain is restored by the bubble contraction. Finally, the shell continues to deform under the action of the water jet and bubble load. However, at this moment, the shell does not reach the threshold of instability, and the shell vibrates due to the bubble pulsation process, and finally approaches a stable state.

The hydrostatic pressure on the shell in a relatively deep-water environment does not reach its load-bearing limit. At this moment, the deformation of the shell is not obvious after the shock wave. The influence of water depth makes the bubble pulsation loading act on the shell more quickly, further increasing the deformation of the shell. Bubble pulsation loading is the key factor inducing the deformation of the shell. When the shell is in an extremely deep-water environment, the hydrostatic pressure on the shell is close to its critical buckling load. Since the prestress in the shell structure is so large, the shell loses its stable state due to the sole action of the explosion shock wave. The water depth for the dynamic buckling of a deep submersible pressure shell, caused by explosion shock wave (2500 m), is about 4.2% smaller than that of quasi-static buckling under hydrostatic pressure (2600 m). Then, the hydrostatic pressure accelerates and amplifies the buckling deformation of the buckled shell. In this case, the explosion shock wave is the main factor inducing shell deformation.

The experiments of Huang et al. [39] further verify the conclusion of this work, showing that underwater shock wave loading is generated by the impact of the impactor. When the external hydrostatic pressure is low, the implosion of the cylindrical shell can be induced by a large impact load. When the hydrostatic pressure is close to the buckling pressure of the cylindrical shell, even a small impact will lead to implosion. The experimental results of Bitter et al. [37] verify that the vibration with mode number greater than 2 can be observed under thinner tubes and higher pressure, meaning it is easier to approach the buckling pressure of cylindrical shells. However, in their work, they only considered the effect of the explosion shock wave and did not consider one of the important parts of underwater explosion loading— bubble pulsation load and water jet impact caused by bubble fluctuation—which is also examined in this work.

Subsequent experiments under real conditions using explosives will be conducted to further study the combined effects of explosion shock waves, bubble pulsation, and collapse. This research aims to further improve the explosion resistance of pressure shells, such as through the use of composite materials, and to provide technical support and a theoretical basis for the application and development of deep-water engineering.

## 5. Conclusions

In this paper, the dynamic response and collapse failure of underwater cylindrical shells were investigated through numerical simulations. The underwater explosion event in a deep-water environment was simulated using the CEL algorithm, with initial hydrostatic pressure included. The influences of water depth and explosion distance on the damage effects of shock wave loading and bubble pulsation were studied. The results show that buckling failure of cylindrical shells can occur in deep-water environments. For near-field explosions, when the explosion distance is smaller than the theoretical maximum bubble radius, the bubble will be attracted to the wall and generate a water jet on the shell wall. The shell collapses due to the combined effects of hydrostatic pressure and the water jet. By contrast, the buckling of cylindrical shells in far-field explosions is mainly triggered by bubble pulsation loading. However, the dominating driving force behind the structure’s eventual collapse is still hydrostatic pressure. For the extreme cases in which the hydrostatic pressure is approaching the critical buckling load capacity of the shell, the shock wave alone can trigger structural instability. Our work demonstrates that the proposed simulation approach can effectively reveal the effects of shock waves, bubble pulsation, and hydrostatic pressure on the failure of target shell structures. It also shows that the triggering mechanisms of the buckling failure of cylindrical shells vary significantly under different combinations of hydrostatic pressure levels and explosion distances. These findings could be used to guide the design of robust marine structures with high survivability against UNDEX.

## Figures and Tables

**Table 1:** Water medium material parameters.

ρ/(kg·m^−3^)	C_0_/(m·s^−1^)	S	γ	Dynamic Viscosity
998	1500	0	0	0.001

**Table 2:** JWL parameters for TNT.

ρ/(kg·m^−3^)	A/GPa	B/GPa	R_1_	R_2_	ω	E/(J·kg^−1^)
1690	371.2	3.21	4.15	0.95	0.3	4,290,000

**Table 3:** Material parameters for 45# steel.

ρ/(kg·m^−3^)	Young’s Modulus/GPa	Poisson’s Ratio	A_1_	B_1_	n	m
7850	200	0.3	263,510,000	130,050,000	0.0915	1

**Table 4:** Comparison between experimental value and simulated value.

Parameter	Experiment [23]	Simulation	Deviation
Maximum bubble radius/mm	79.1	77.0	2.65%
Bubble pulsation period/ms	6.0	6.4	6.67%

## Data Availability

The raw data supporting the conclusions of this article will be made available by the authors on request.
